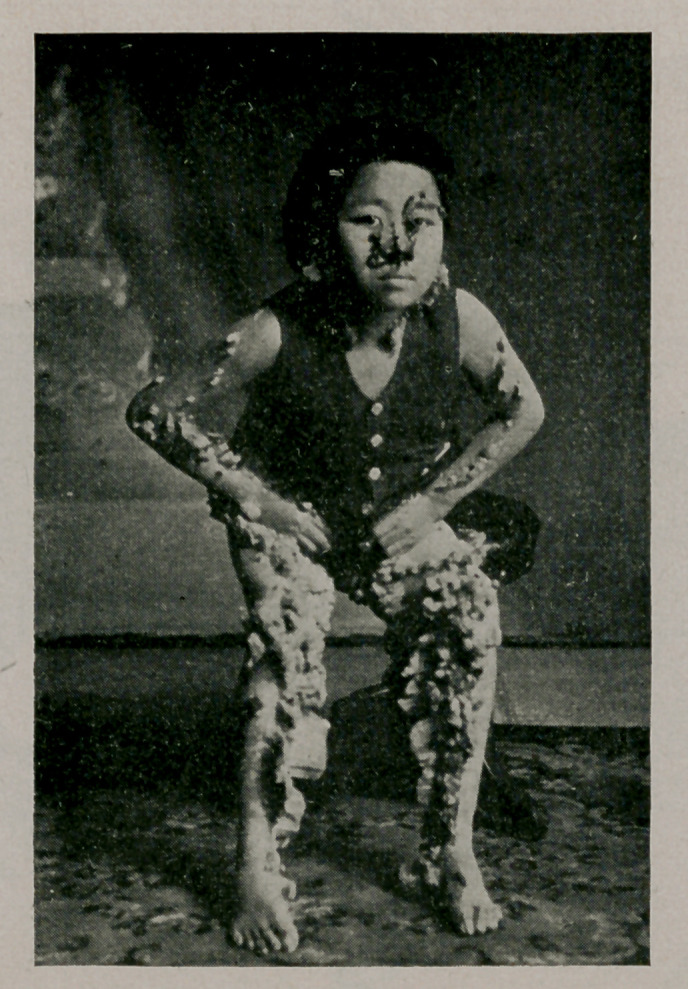# Case of Cutaneous Horns

**Published:** 1915-01

**Authors:** 


**Case of Cutaneous Horns.** Male Korean, aged 19. There is no family history. Immediately after birth red spots were noticed on the arm, leg, chin, forehead, nose and about the umbilicus. When two years old, warts began to appear in these places. The horns cause no pain, unless pulled as by the clothing, but itching is felt in rainy or windy weather and meat causes urticaria. He prefers to stay where it is cool.

## Figures and Tables

**Figure f1:**
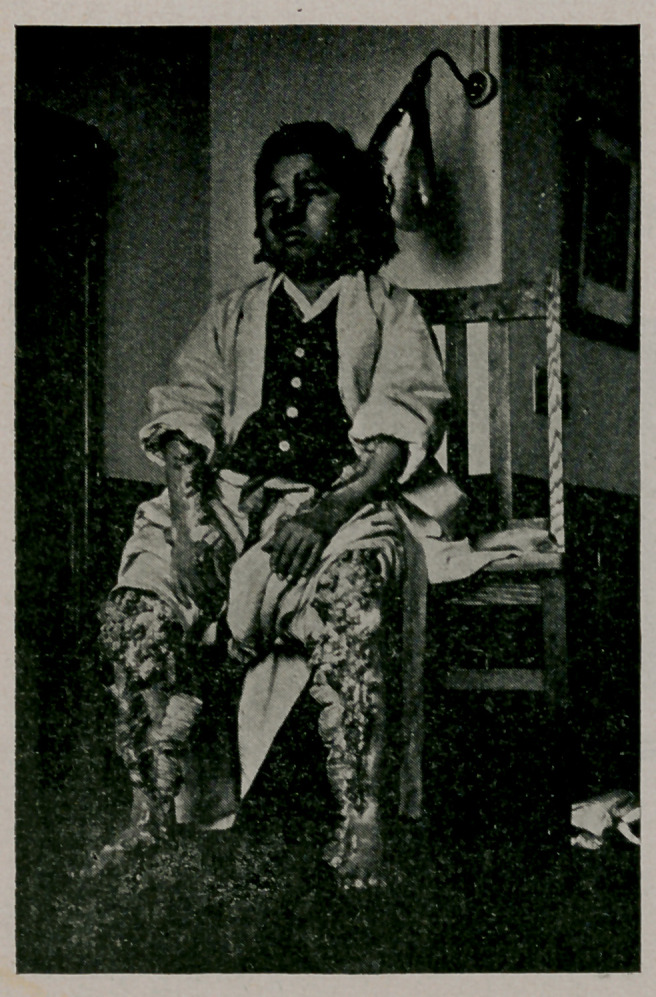


**Figure f2:**